# Relationship between location and activity in injurious falls: an exploratory study

**DOI:** 10.1186/1471-2318-10-40

**Published:** 2010-06-18

**Authors:** Michel HC Bleijlevens, Joseph PM Diederiks, Marike RC Hendriks, Jolanda CM van Haastregt, Harry FJM Crebolder, Jacques ThM van Eijk

**Affiliations:** 1 Department of Health Care and Nursing Science, Faculty of Health, Medicine and Life Sciences Maastricht University, PO box 616, 6200 MD Maastricht, The Netherlands; 2 School for Public Health and Primary Care (Caphri), Faculty of Health, Medicine and Life Sciences, Maastricht University, PO box 616, 6200 MD Maastricht, The Netherlands; 3 Department of Healthcare studies, Faculty of Health, Medicine and Life Sciences Maastricht University, PO box 616, 6200 MD Maastricht, The Netherlands; 4 Department of Movement Science, Faculty of Health, Medicine and Life Sciences Maastricht University, PO box 616, 6200 MD Maastricht, The Netherlands; 5 Department of Health Organization Economics and Policy, Faculty of Health, Medicine and Life Sciences Maastricht University, PO box 616, 6200 MD Maastricht, The Netherlands; 6 Department of General Practice, Faculty of Health, Medicine and Life Sciences, Maastricht University, Maastricht, The Netherlands; 7 Department of Social Medicine, Faculty of Health, Medicine and Life Sciences Maastricht University, PO box 616, 6200 MD Maastricht, The Netherlands

## Abstract

**Background:**

Knowledge about the circumstances under which injurious falls occur could provide healthcare workers with better tools to prevent falls and fall-related injuries. Therefore, we assessed whether older persons who sustain an injurious fall can be classified into specific fall types, based on a combination of fall location and activity up to the moment of the fall. In addition, we assessed whether specific injurious fall types are related to causes of the fall, consequences of the fall, socio-demographic characteristics, and health-related characteristics.

**Methods:**

An exploratory, cross-sectional study design was used to identify injurious fall types. The study population comprised 333 community-dwelling Dutch elderly people aged 65 years or over who attended an accident and emergency department after a fall. All participants received a self-administered questionnaire after being discharged home. The questionnaire comprised items concerning circumstances of the injurious fall, causes of the fall, consequences of the fall, socio-demographic characteristics and health-related characteristics. Injurious fall types were distinguished by analyzing data by means of HOMALS (homogeneity analysis by means of alternating least squares).

**Results:**

We identified 4 injurious fall types: 1) Indoor falls related to lavatory visits (hall and bathroom); 2) Indoor falls during other activities of daily living; 3) Outdoor falls near the home during instrumental activities of daily living; 4) Outdoor falls away from home, occurring during walking, cycling, and shopping for groceries. These injurious fall types were significantly related to age, cause of the fall, activity avoidance and daily functioning.

**Conclusion:**

The face validity of the injurious fall typology is obvious. However, we found no relationship between the injurious fall types and severity of the consequences of the fall. Nevertheless, there appears to be a difference between the prevalence of fractures and the cause of the fall between the injurious fall types. Our data suggests that with regard to prevention of serious injuries, we should pay special attention to outdoor fallers and indoor fallers during lavatory visits. In addition, we should have special attention for causes of the fall. However, the conclusions reached in this exploratory analysis are tentative and need to be validated in a separate dataset.

## Background

Falls and fall-related injuries in the elderly constitute a significant problem for individuals as well as for society. One out of three elderly persons aged 65 years or older falls at least once a year [1-3]. In half of all cases, a fall results in some kind of physical injury [4-6]. Approximately 5% of all falls in community-dwelling elderly people result in a fracture. Another 5 to 10% of falls result in serious soft tissue injury, such as severe head injury and joint dislocations [3,4,7-12]. In addition, falls can have considerable psychosocial consequences, like fear of falling, activity avoidance, and social isolation [13,14]. However, due to variations in the definitions and methods of measuring falls it is difficult to compare the outcomes of different studies [15].

Falls resulting in injuries require special attention, since these falls are responsible for increased levels of healthcare utilization and consequent costs [6,16-21]. Unless we undertake effective preventive measures, the societal and economic burden of falls and fall-related injuries will increase in the coming decades as a result of the growing number of aged people. It therefore seems important to develop fall prevention measures to reduce injurious falls.

In recent decades, many interventions have been developed to prevent falls in older persons [22]. Prevention programmes comprising multidisciplinary and multifactorial interventions that screen for health and environmental risk factors and address these factors are expected to be particularly effective in preventing falls [1-3,22-25]. Nevertheless, systematic reviews provide only modest benefit of multifactorial programs in preventing falls [1-3,23-25]. Interventions to prevent fall-induced injuries, often aim to reduce the risk of fractures by taking single intervention measures like regular exercise, intake of nutritional supplements (calcium, vitamin D) or the use of hip protectors [3,23,26]. However, evidence for the effectiveness of these interventions is even more limited [3,23]. Therefore, we need to search for additional strategies to improve the effectiveness of these interventions. We should especially think of strategies to ensure less fall-related injuries if a fall does occur. For example, it may be useful to use energy-absorbent surfaces in high risk locations and hip protectors (injury-site protection) in order to decrease the impact of a fall. However, to be able to do this, we need insight in the circumstances of injurious falls. Knowledge about the circumstances under which injurious falls occur could provide healthcare workers with better tools to prevent falls and fall-related injuries. Several studies already reported on circumstances under which falls occur, such as the location of the fall and the activity the person was engaged in up to the moment of the fall. However, these studies did not assess the combination of location and activity prior to the fall [9,27-33]. Therefore, the present study aims to answer the following questions:

1. Is it possible to establish a classification of injurious fall types based on fall location and activity up to the moment of the fall?

2. What is the relationship between injurious fall types on the one hand and socio-demographic characteristics, causes of the fall, consequences of the fall, and health-related characteristics on the other?

## Methods

### Design, participants, and setting

We carried out an exploratory, cross-sectional study to identify injurious fall types based on location of the fall and activity up to the moment of the fall. The population of this study was derived from a randomized controlled trial (RCT) assessing the effectiveness and cost-effectiveness of a multidisciplinary fall prevention programme [34,35]. Injurious falls were defined as falls resulting in some kind of physical injury for which persons attended the Accident & Emergency (A&E) department. The study design and protocols were approved by the Medical Ethics Committee of Maastricht University and the University Hospital Maastricht. Eligible persons were community-dwelling elderly people aged 65 years and over living in Maastricht (the Netherlands) or its surrounding area. All persons had visited the A&E department at the University Hospital Maastricht (which includes an out-of-hours GP service) for the consequences of a fall. Eligible persons were excluded if they were unable to communicate in Dutch, unable to complete questionnaires or interviews by telephone, cognitively impaired (a score of less than 4 on the Abbreviated Mental Test 4), admitted to a hospital or other institution for more than four weeks from the date of inclusion, permanently bedridden or fully dependent on a wheelchair. A total of 333 persons were included in the present study.

### Measurements

All participants received a self-administered questionnaire after being discharged home (i.e. immediately after treatment of the injuries resulting from the fall or after a period of hospitalization). The mean time between the fall for which the participants visited the A&E department at the University Hospital Maastricht and completing the questionnaire was 1.6 months (SD = 0.55). The questionnaire comprised the following items:

• *Circumstances of the injurious fall: *location of the fall and the person's activity up to the moment of the fall. Participants were asked to indicate where they were at the moment they fell and if they could indicate what they were doing. Participants could choose from a list of thirteen pre-defined locations and nine pre-defined activities, or describe other locations and activities up to the moment of the fall. Two researchers (MB and JD) independently reviewed the answers to these two questions and classified the answers into two variables, fall location (n = 10 categories) and activity (n = 9 categories). Disagreements were resolved by consensus or by consulting a third party (MH).

• *Causes of the fall: *self-reported perceived cause of the fall. Participants were asked what, in their opinion, was the cause of their fall. They could choose from a list of thirteen pre-defined causes or describe other possible causes of their fall(s). More than one cause could be indicated. Two researchers (MB and MH) independently reviewed the answers to this question and classified the answers into two variables (intrinsic and extrinsic cause) based on two previous studies [13,19]. Disagreement was resolved by consensus or by consulting a third party (JD). The reported cause of a fall could be intrinsic, extrinsic, a combination of intrinsic and extrinsic, or unknown.

• *Consequences of the fall: *fear of falling (1 item, five-point Likert scale); activity avoidance due to fear of falling (1 item, five-point Likert scale), recuperation from the fall (1 item, five-point Likert scale); severity of the injury, defined as major or minor injury. Fractures, joint dislocations, and lacerations requiring sutures were considered major injuries. Lacerations without sutures, bruises, abrasions, sprains, and other minor soft tissue injuries were considered minor injuries. This classification is in accordance with the definition of major and minor injuries reported by Nevitt and colleagues [9]. We asked a GP (HC) to assess all injuries that did not fit the definitions we used and to classify them into major or minor injury.

• *Socio-demographic characteristics: *age; gender; living situation (living alone versus not living alone); level of education (primary school or less versus more than primary school).

• *Health-related characteristics: *health complaints (19 items), perceived health (first item of the RAND-36) [36], daily functioning (Frenchay Activities Index, FAI). The FAI measures participation in social and instrumental daily living activities and comprises 15 items covering three dimensions: domestic chores; work/leisure; and outdoor activities. Individual item responses capture frequency of participation ranging from 0 (never or none) to 3 (daily or weekly). Summary scores are derived by adding the items, with scores ranging from 0 (no activity) to 45 (very high participation) [37]; activities of daily living disability (ADL subscale of the Groningen Activity Restriction Scale, GARS). This subscale measures disability in the domain of personal care and comprises 11 items. The items refer to what respondents are able to do and not to their actual performance. The theoretical minimum is 11, indicating the absence of disability and the theoretical maximum 44, indicating that a person is highly disabled[38].

### Statistics

SPSS statistical software (version 13) was used for analyses. Injurious fall types were distinguished by analyzing data about fall location and activity up to the moment of the fall by means of HOMALS (homogeneity analysis by means of alternating least squares). HOMALS quantifies the nominal variables fall location (10 answer categories) and activity (9 answer categories) by assigning numerical values to each answer category of the two variables and to each person in the study. HOMALS identifies associations between fall location and activity in a two-dimensional plot. The outcome figure represents coordinates for every single person based on location and activity (participant scores). Coordinates of persons with different answer patterns are positioned far apart, whereas persons with similar answer patterns are positioned in relatively close proximity. Persons who are located closely together in the plot constitute a homogeneous group. In this way were are able to identify injurious fall types [39].

If injurious fall types were identified we further investigated the relation between these injurious fall types on the one hand and socio-demographic characteristics, perceived cause of the fall, consequences of the fall, and health-related characteristics on the other by means of chi-square (α = 0.05) and one-way ANOVA with Tukey's criterion for post-hoc pairwise comparisons (α = 0.05).

## Results

### Circumstances of the falls

Table 1 shows the distribution of the fall locations. The majority of falls occurred outside the home. The location where most of the falls occurred was the street or sidewalk (38%).

**Table 1:** Distribution of fall locations (n = 333)

*Location*	*Number*	*(%)*
**Indoor locations (own home)**		
Stairs	36	(10.8)
Living room and studio at home	31	(9.3)
Bedroom	18	(5.4)
Hallway	18	(5.4)
Bathroom	14	(4.2)
Kitchen and cellar	12	(3.6)
**Indoor locations (away from home)**		
Shop, post office, church, bar, etc	19	(5.7)
**Outdoor locations around one's home**		
Access path, garden	35	(10.5)
Other (balcony, terrace)	3	(0.9)
**Outdoor locations away from home**		
Street or sidewalk, park, forest, pasture, playground, etc	147	(44.1)
***Total***	***333***	***(100.0)***

Table 2 shows the activities up to the moment of the fall. Not surprisingly, walking was the most prevalent activity up to the moment of a fall (21%). A substantial proportion of the falls was mobility-related (about 45%), while about 20% were related to household activities.

**Table 2:** Distribution of activities up to the moment of the fall (n = 333)

*Activity*	*Number*	*(%)*
Instrumental activities of daily living (IADL)	75	(22.5)
Walking	71	(21.3)
Catching and moving things	51	(15.3)
Activities of daily living (ADL)	33	(9.9)
Lavatory visit	22	(6.6)
Cycling	19	(5.7)
Social activities (for example: visiting friends or family or voluntary work)	16	(4.8)
Climbing stairs	9	(2.7)
Other	37	(11.1)
***Total***	***333***	***(100.0)***

### Types of injurious falls

**Figure 1:**
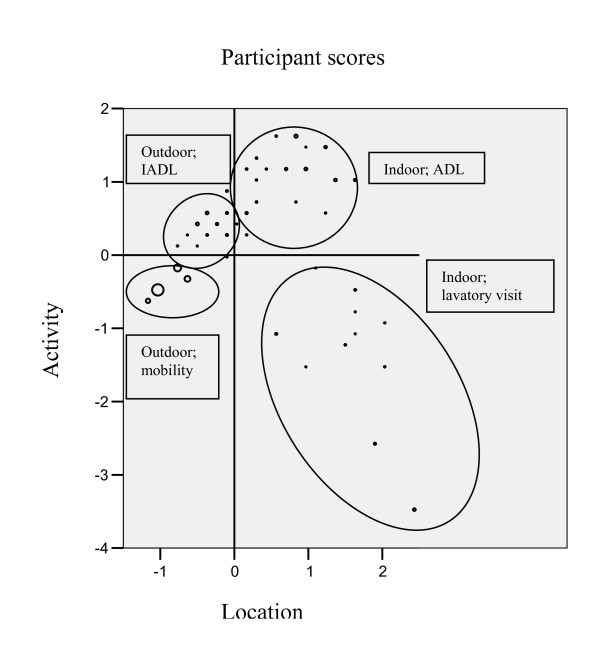
**Injurious Fall Types in HOMALS Plot of Participant Scores**. Figure 1 shows the optimal quantifications for both the location of the fall and the activity up to the moment of the fall, and reveals four types of injurious falls. The size of the dots represents the number of participants; the bigger a dot, the more participants it represents.

Figure 1 shows the distribution of persons within the two-dimensional HOMALS solution. It reduced the complexity of the available data, and yielded a two-dimensional solution with eigenvalues of 0.879 and 0.752 for the first and second dimension, respectively.

The first dimension represents the fall location ranging from outdoors (away from own home and around one's home) to indoors (indoor locations away from one's home and indoor in one's home (kitchen/cellar, stairs, living room/studio at home, hallway, bedroom, bathroom). The second dimension represents the activities and ranges from lavatory visit, through outdoor activities (cycling, walking, social activities) to indoor activities (IADL, ADL, catching and moving things, and ascending and descending stairs).

We identified a group of injurious falls occurring in the bathroom/hall during lavatory visit (group 1), which is opposed to a group of outdoor falls during walking, cycling, and shopping (group 4). Furthermore, we distinguished a group of indoor falls during ADL (group 2) and a group outdoor falls around the respondents' home (garden) during IADL (group 3). This last group is located at the transition between outdoor locations and indoor locations. Based on these four groups of injurious falls, we defined the following four injurious fall types:

1. Indoor falls in the hall and bathroom, predominantly during lavatory visit

2. Indoor falls (at other locations than the hall and bathroom), predominantly during ADL

3. Outdoor falls near the home (garden, access path), predominantly during IADL

4. Outdoor falls away from home, occurring predominantly during walking, cycling, and shopping for groceries

### Perceived causes and consequences of the fall

The majority of the 333 respondents reported an extrinsic cause of their fall (n = 169, 51%), whereas 112 respondents (34%) reported an intrinsic cause of their fall. A total of 36 respondents (11%) stated that the cause of their fall was a combination of intrinsic and extrinsic causes. One hundred and eighty respondents had sustained a fall resulting in a major injury (54%). Fractures had occurred in 121 of the 333 respondents who sustained an injurious fall (36%). About two third of the respondents experienced some fear of falling (n = 226), and about half (n = 183) avoided activities because they were afraid to fall during these activities. Recuperation after the fall was judged reasonable to good by 236 respondents (71%).

### Socio-demographic characteristics

All of the 333 participants were community-dwelling and ranged in age from 65 to 95 years, with a mean age 74.9 (SD 6.4). The majority of the study population was living with a partner at the time of the fall (77%), had higher than primary school education (72%), and was female (69%).

### Health-related characteristics

The 333 respondents had an average of 6 health complaints (SD 4.1) and had mean scores on the FAI and GARS of 23.5 (SD 8.7) and 17.2 (SD 6.7), respectively. A total of 302 (91%) persons rated their health as good to excellent.

### Relationship between fall types and other characteristics

Table 3 shows that intrinsic causes of falls were significantly more frequent for indoor than for outdoor locations (types 1 and 2 versus types 3 and 4). Moreover, type 4 fallers reported significantly more extrinsic causes than fallers in the other injurious fall types. We found no relationship between injurious fall type and the consequences of the fall, except for activity avoidance (p = 0.044). We found that persons who were younger than were predominantly involved in type 4 falls (table 4). Table 5 shows a number of significant differences in health-related characteristics between the four injurious fall types. We found a significant difference between type 3 and type 4 falls and between type 1 and type 4 falls in terms of the total number of health complaints. Type 4 fallers reported less health complaints. As regards the total FAI score, there was a significant difference between types 1 and 2 and between types 1 and 4. Type 1 fallers had less favourable scores on the FAI. Finally, the GARS score was significantly different between type 3 and type 4 falls and between type 1 and type 4 falls. Type 4 fallers had more favourable scores on the GARS.

**Table 3:** Relationship of causes and consequences of the fall with injurious fall types

	Type 1^* ^**Number (%)**	**Type 2**^**† **^**Number (%)**	**Type 3**^‡ ^**Number (%)**	**Type 4**^§ ^**Number (%)**	P-value
**Distribution of participants within fall types**	32 (9.6)	116 (34.8)	38 (11.4)	147 (44.1)	
					
**Causes of the fall**					**0.000**
Intrinsic cause	21 (18.8)	49 (43.8)	13 (11.6)	29 (25.9)	
Extrinsic cause	3 (1.8)	50 (29.6)	20 (11.8)	96 (56.8)	
					
**Consequences**					
Injury (major injury versus minor injury)					0.622
% Major injury	16 (8.9)	58 (32.2)	22 (12.2)	84 (46.7)	
%Minor injury	16 (10.5)	58 (41.2)	16 (10.5)	63 (41.2)	
Injury (fracture versus no fracture)					0.172
%Fracture	12 (9.9)	33 (27.3)	15 (12.4)	61 (50.4)	
% No fracture	20 (9.4)	83 (39.2)	23 (10.8)	86 (40.6)	
Recuperation from the fall					0.755
%≥reasonable	21 (8.9)	83 (35.2)	25 (10.6)	107 (45.3)	
%≤moderate	11 (11.3)	33 (34.0)	13 (13.4)	40 (41.2)	

*Type 1: Indoor falls in the hall and bathroom, during lavatory visit

†Type 2: Indoor falls (at other locations than the hall and bathroom), during ADL

‡Type 3: Outdoor falls near the home, predominantly during IADL

§Type 4: Outdoor falls away from home, occurring during mobility-related activities

Row totals add up to 100% for each of the categories listed

**Table 4:** Relationship of socio-demographic characteristics and health-related characteristics with injurious fall types

	Type 1^* ^**Number (%)**	**Type 2**^**† **^**Number (%)**	**Type 3**^‡ ^**Number (%)**	**Type 4**^§ ^**Number (%)**	P-value
**Distribution of participants within fall types**	32 (9.6)	116 (34.8)	38 (11.4)	147 (44.1)	
					
**Socio-demographic characteristics**					
Age					**0.036**
% <80 year	22 (8.6)	82 (32.0)	28 (10.9)	124 (48.4)	
% ≥80 year	10 (13.0)	34 (44.2)	10 (13.0)	23 (29.9)	
Gender					0.121
% Female	21 (9.2)	80 (35.1)	20 (8.8)	107 (46.9)	
% Male	11 (10.5)	36 (34.3)	18 (17.1)	40 (38.1)	
Living situation					0.850
% Living alone	14 (9.7)	48 (33.3)	15 (10.4)	67 (46.5)	
% Living with a partner	18 (9.6)	68 (36.2)	23 (12.2)	79 (42.0)	
Level of education					0.748
% ≤primary school	10 (10.6)	33 (35.1)	13 (13.8)	38 (40.4)	
% >primary school	22 (9.2)	83 (34.7)	25 (10.5)	109 (45.6)	
					
**Health-related characteristics**					
Fear of falling					0.981
% ≥sometimes	22 (9.7)	80 (35.4)	26 (11.5)	98 (43.4)	
% ≤almost never	10 (9.3)	36 (33.6)	12 (11.2)	49 (45.8)	
Activity avoidance					**0.044**
% ≥sometimes	20 (10.9)	71 (38.8)	24 (13.1)	68 (37.2)	
% ≤almost never	12 (8.0)	45 (30.0)	14 (9.3)	79 (52.7)	
Perceived health (≥good)					0.546
% ≥good	31 (10.3)	105 (34.8)	33 (10.9)	133 (44.0)	
% ≤moderate	1 (3.2)	11 (35.5)	5 (16.1)	14 (45.2)	

*Type 1: Indoor falls in the hall and bathroom, during lavatory visit

†Type 2: Indoor falls (at other locations than the hall and bathroom), during ADL

‡Type 3: Outdoor falls near the home, predominantly during IADL

§Type 4: Outdoor falls away from home, occurring during mobility-related activities

Row totals add up to 100% for each of the categories listed

**Table 5:** ANOVA of health-related characteristics and injurious fall types

	Type 1^* ^**(n = 32)**	**Type 2**^**† **^**(n = 116)**	**Type 3**^‡ ^**(n = 38)**	**Type 4**^§ ^**(n = 147)**	P-value (ANOVA)	P-value
**Total health complaints**	7.75	6.34	8.26	5.29	0.000	0.010 (types 1 and 4) 0.000 (types 3 and 4)
**Total FAI**^|| ^**score (0-45) **^#^	18.94	23.32	21.61	25.03	0.001	0.050 (types 1 and 2) 0.002 (types 1 and 4)
**Total GARS**^¶ ^**score (11-44) **^#^	20.16	17.36	19.58	15.90	0.001	0.010 (types 1 and 4) 0.000 (types 3 and 4)

*Type 1: Indoor falls in the hall and bathroom, during lavatory visit

†Type 2: Indoor falls (at other locations than the hall and bathroom), during ADL

‡Type 3: Outdoor falls near the home, predominantly during IADL

§Type 4: Outdoor falls away from home, occurring during mobility-related activities

^||^Frenchay Activities Index; ^¶^Groningen Activity Restriction Scale; ^#^the underlined score is the most favourable score

## Discussion

The circumstances under which injurious falls occur have been accurately described in previous studies [9,27-32]. Although fall location and activity were the most common reported circumstances in these studies, none of the studies assessed whether persons sustaining injurious falls can be classified into specific fall types based on a combination of fall location and activity up to the moment of the fall. By doing so we identified 4 injurious fall types in the present study:

1. Indoor falls in the hall and bathroom, predominantly during lavatory visits

2. Indoor falls (at other locations than the hall and bathroom), predominantly during ADL

3. Outdoor falls near the home (garden, access path), predominantly during IADL

4. Outdoor falls away from home, occurring predominantly during walking, cycling, and shopping for groceries

We concluded that type 1 fallers (indoor fallers in the hall and bathroom during lavatory visits) proved to belong to the most inactive group (lowest FAI score), having more problems coping with activities of daily living (highest GARS score). Type 4 fallers (persons who experienced a fall away from home during mobility-related activities) predominantly were younger (aged <80), more active and have the most favourable daily functioning (GARS) scores. This group seems to consist of those elderly people who are less frail and still venture outside. A recent study confirms that persons who are less frail are engaged in productive activity [40]. We did not find a significant difference between injurious fall types in terms of the consequences of the fall, except for activity avoidance after the fall. Indoor falls, with the exception of those in the hall and bathroom during ADL (type 2 fallers) led to fewer fractures than the other fall types (approximately 10%). It has been suggested that indoor falls carry a lower risk of injury, because indoor surfaces may be more absorbing than outside ones [9], because persons who fall inside the house are more likely to fall on carpeted floors. Our data tend to support this suggestion. In addition, indoor falls may also carry a lower risk of injury because activities resulting in a fall inside the house may be less vigorous than activities resulting in a fall outside the house, and therefore create less force at fall impact.

Our finding that a majority of the injurious falls took place outdoors is consistent with previous reports [5,29-31]. Walking accounted for the largest proportion of the activities respondents were engaged in, as was also reported from previous studies [5,30,31,41]. The younger age group was more often engaged in leisure activities and sustained more outdoor falls. The more frail older persons in our study tended to stay in their own house and predominantly fell during ADL and particularly during lavatory visits. These findings resemble the findings of previous studies, which found that vigorous persons were more likely to fall outside the home during displacement activities such as climbing ladders or engaging in sports, while frail older persons fell during routine daily activities at home [42-44].

The present study has some limitations. First, all subjects in our sample sustained an injurious fall and attended the A&E department of a hospital to get treatment for the consequences of their injurious falls. We did not include persons who visited their GP with the consequences of an injurious fall. Moreover, we also did not select those persons who did not seek medical attention at all for the consequences of the injurious fall. Therefore it is likely that injuries after a fall, as represented in our population, are more serious compared to the injuries after a fall in a more general population of older adults. Second, all data were self-reported. Although the accuracy of self-report data remains unclear, older people are often the only witnesses of their fall events, so self-reports remain an important source of information about falls [45]. Third, it should be noted that that the analyses are data-driven, meaning that there was no a priori hypotheses formulated. HOMALS was allowed to come up with the best partitioning between the four fall types.

## Conclusion

In conclusion, we succeeded in classifying injurious falls based on fall location and activity up to the moment of the fall. The face validity of the injurious fall typology is obvious. However, we did not find any relationship between the four injurious fall types and severity of the consequences of the fall. Nevertheless, although not significant, there appears to be a difference between the prevalence of fractures between the injurious fall types. Outdoor falls and indoor falls related to lavatory visits resulted in more fractures, compared with indoor falls during ADL. This may indicate that with regard to the prevention of serious injuries, we should pay special attention to outdoor fallers and indoor fallers during lavatory visits. In addition, there seems to be a difference in fall location and activity up to the moment of the fall between the younger and more active elderly, who still go outdoors, and the more frail older people who tend to stay indoors. Those persons who fell outdoors predominantly reported an extrinsic cause of their fall, whereas those persons who fell indoors reported an intrinsic cause. Our data suggests that in case of a faller (<80 year) who has fallen outside and a faller (≥80 year) who has fallen inside we should have special attention for extrinsic causes and intrinsic causes, respectively. However, it is recognised more and more that falls are the consequence of the interaction between a number of risk factors, both intrinsic and extrinsic [10,11,32,46,47]. Therefore, the conclusions reached in this exploratory analysis are tentative and need to be validated in a separate dataset.

## Competing interests

The authors declare that they have no competing interests.

## Authors' contributions

JD and MB developed the study design with input form all other authors. MB and MH coordinated the data collection and conducted the statistical analyses with input from JD. JH, HB and JE provided methodological input. MB drafted the manuscript with input from the other authors. All authors read and approved the final manuscript.

## Pre-publication history

The pre-publication history for this paper can be accessed here:

http://www.biomedcentral.com/1471-2318/10/40/prepub
